# A Multifactorial Case of Acquired Hemophilia A

**DOI:** 10.7759/cureus.101728

**Published:** 2026-01-17

**Authors:** Jason P Willis, Timothy Kanne, Alex Cole, Grant Nelson

**Affiliations:** 1 Medicine, Edward Via College of Osteopathic Medicine, Auburn, USA; 2 Internal Medicine, Edward Via College of Osteopathic Medicine, Auburn, USA; 3 Medicine, East Alabama Medical Center, Opelika, USA

**Keywords:** acquired hemophilia a (aha), aquired hemophilia, hematoma spontaneous, lupus anticoagulant, multifactorial

## Abstract

Acquired hemophilia A (AIHA) is a rare autoimmune bleeding disorder in which the body develops autoantibodies against factor VIII, leading to spontaneous bleeding. It has an incidence of about one person per million each year. AIHA carries high risks; these symptoms are only exacerbated with other comorbidities, as explored in this patient, a 73-year-old female patient with a history of heart disease, diabetes, hypertension, and a positive lupus anticoagulant (LA) test. She presented with a rapidly growing neck hematoma that compromised her airway, requiring immediate intubation and admittance to the ICU. Laboratory tests showed a severely prolonged activated partial thromboplastin time (aPTT) and critically low factor VIII levels, confirming the diagnosis of AIHA. Despite early treatment with corticosteroids, cyclophosphamide, rituximab, and recombinant factor VIIA, she experienced repeated bleeding episodes and developed complications such as neutropenic fever and refractory depression. Ultimately, due to the cumulative impact of these complications, she was transitioned to palliative care and died. This case highlights the importance of prompt diagnosis and treatment, the difficulties of balancing immunosuppression and bleeding risk, and the impact significant illnesses can have on mental health. Early recognition, multidisciplinary management, and individualized treatment strategies are crucial to improving outcomes in patients with this rare disorder.

## Introduction

Acquired hemophilia A (AIHA) is a rare disorder characterized by the development of antibodies against factor VIII (FVIII), leading to the inhibition of FVIII and spontaneous or excessive bleeding. The incidence of AIHA is approximately one per million per year and has a mortality rate of around 20% despite appropriate treatment [1]. Unlike congenital hemophilia, AIHA occurs in patients with no prior history of bleeding disorders. It is often associated with pregnancy, autoimmune diseases, malignancies, infections, or the use of certain drugs, although nearly half of the cases are idiopathic [1,2]. Clinically, AIHA commonly presents with spontaneous hemorrhages in soft tissues, muscles, or mucosal surfaces, whereas hemarthrosis, which is typical of congenital hemophilia A, is rare [1,2]. Diagnosis is based on the detection of a prolonged activated partial thromboplastin time (aPTT) that fails to correct in mixing studies, as well as reduced FVIII activity and the presence of FVIII inhibitors [1,2]. Management includes controlling bleeding with bypassing agents, such as recombinant activated factor VII (rFVIIa) or activated prothrombin complex concentrate (aPCC), and immunosuppressive therapy, typically with corticosteroids and cyclophosphamide [1,2]. Emerging treatments, such as emicizumab, offer a promising alternative to traditional immunosuppressive regimens and superior bleeding prophylaxis [3,4].

In this case, we present a 73-year-old female patient with multiple comorbidities, including coronary artery disease, type 2 diabetes mellitus, hypertension, subclavian artery stenosis, heart failure with preserved ejection fraction, and a known lupus anticoagulant (LA) who developed a rapidly enlarging neck hematoma, which compromised her airway. Further workup revealed severe FVIII deficiency with persistent coagulopathy, confirming the diagnosis of AIHA. Despite aggressive immunosuppressive therapy and factor replacement, her condition deteriorated, and she suffered multiple complications, ultimately leading to a transition to palliative care.

This case highlights the importance of diagnosing and managing AIHA as well as the challenges of balancing bleeding and thrombotic risks, the complications associated with immunosuppressive therapy, and the profound impact mental health can have on a patient’s prognosis.

## Case presentation

**Figure 1:**
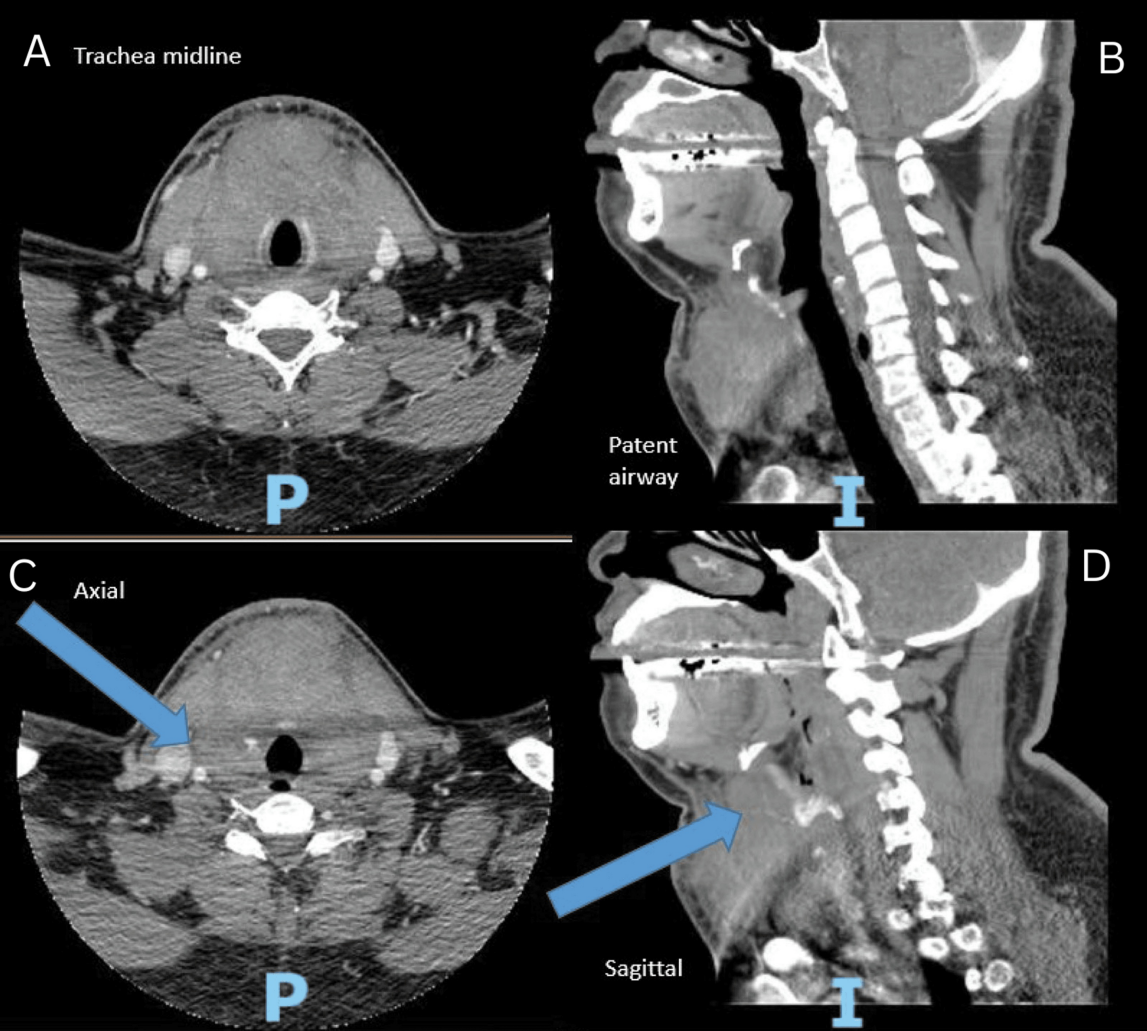
Example of large neck hematoma (A) Axial view of hematoma. (B) Sagittal view of hematoma. (C) Axial view of hematoma with blue reference arrow pointing toward the hematoma. (D) Sagittal view of hematoma with blue reference arrow pointing toward the hematoma

A 73-year-old female patient with a past medical history of coronary artery disease, type 2 diabetes mellitus, hypertension, mitral regurgitation, hyperlipidemia, subclavian artery stenosis, heart failure with preserved ejection fraction, and LA positive was admitted to the intensive care unit (ICU) due to a rapidly enlarging neck hematoma (Figure 1), which led to airway instability and necessitated intubation. The patient had recurrent upper extremity deep venous thromboses (DVTs) during their last admission and was being worked up for positive LAs in the outpatient setting.

Initial laboratory workup demonstrated hemoglobin and hematocrit (H&H) levels of 6.9 g/dL and 19.1%, respectively. Coagulation studies revealed a prolonged aPTT of 101 seconds and a prothrombin time (PT) of 9.2 seconds. A mixing study failed to correct the prolonged PTT, and testing confirmed a dilute Russell’s viper venom time (DRVVT) of 33. Labs are summarized in Table 1. 

**Table 1:** Patient labs PT: prothrombin time; PTT: partial thromboplastin time; DRVTT: dilute Russell's viper venom time; WBC: white blood cell

Lab	Measured value day 1	Measured value day 21	Measured value day 41	Measured value day 58	Measured value day 62	Reference range
Hemoglobin	6.9	8.3	9.8	-	-	12-16 g/dL
Hermatocrit	19.1	-	27.6	-	-	36-45%
PTT	101	68	60	-	-	25-40 seconds
PT	9.2	-	13	-	-	10-13 seconds
DRVTT	33	-	-	-	-	<1.20 ratio
Factor VIII	<1%	-	Undetectable	1%	4%	50-150% of average
Na	-	-	112	-	-	136-142 mEq/L
WBC	-	-	0.7	-	-	4.5-11.0 x 10^3^ cells/mm^3^
Blood culture	-	-	-	No growth	-	No growth

Given suspected AIHA, the hematology/oncology team was consulted, who started the patient on immunosuppressive therapy consisting of corticosteroids, cyclophosphamide, and rituximab. Factor VIII activity was <1%. Over the following days, the patient showed gradual clinical improvement and was extubated on day six and transferred to a step-down unit. She switched to oral cyclophosphamide at 2 mg/kg daily with a tapering prednisone regimen, and two doses of recombinant activated factor VIIA were administered. She was discharged on day 21 with an improved aPTT of 68 and a hemoglobin of 8.3 g/dL.

The patient was readmitted 20 days later with neutropenic fever, which was suspected to be secondary to cyclophosphamide-induced myelosuppression. Therefore, this was discontinued. Empiric antibiotic therapy was started, including vancomycin, cefepime, and metronidazole. Laboratory evaluation demonstrated an aPTT of 60 seconds, PT of 13 seconds, H&H of 9.8 g/dL and 27.6%, respectively, serum sodium of 112 mEq/L, undetectable Factor VIII levels, and a WBC of 0.7 × 10⁹/L. Blood cultures remained negative throughout the admission. In place of cyclophosphamide, prednisone was initiated. During hospitalization, the patient developed encephalopathy, which improved with a switch from cefepime to levofloxacin. On day 17, Factor VIII activity had increased to 1%. Because of this and an improved hemoglobin, cyclosporine was initiated. She was discharged on this regimen a day later.

Three days after the second discharge, the patient was readmitted with an acute gastrointestinal bleed, which was suspected to be a consequence of the cyclosporine. Given the risk of worsening bleeding, both prednisone and cyclosporine were withheld. Factor VIII activity was measured at 4%, and the aPTT was normal. By day four, Factor VIII levels had increased to 7%. However, during hospitalization, the patient developed significant psychiatric symptoms, including major depression with borderline catatonia, which was attributed to her self-perceived prognosis and other comorbidities. Consequently, dextroamphetamine and bupropion were added. Several days later, her hemoglobin stabilized, but she was kept in the hospital due to persistent depressive symptoms. 

On day 35, she was transferred to a psychiatric unit for further evaluation and management. Over time, the patient exhibited a progressive decline in functional status, marked by worsening global weakness, refractory depression, inadequate oral intake, dysphagia, and persistent hyponatremia secondary to syndrome of inappropriate antidiuretic hormone secretion (SIADH). Despite multiple interventions, her condition continued to deteriorate. On day 37, a decision was made to transition her to palliative care. Her condition progressively declined, and she ultimately died on day 54 of the third admission.

## Discussion

At first glance, it may appear that the patient’s positive LA contributed to her acquiring hemophilia A. Though there are select cases of LA and factor VIII (FVIII) inhibitors persisting simultaneously [5], the presence of LA and FVIII inhibitors can complicate the interpretation of coagulation assays. Both conditions can lead to a prolonged APTT, making it challenging to distinguish between them. Studies have shown that LA can interfere with assays designed to detect FVIII inhibitors, potentially leading to false-positive results [6,7]. For instance, Sahud et al. demonstrated that patient samples positive for LA frequently yielded false-positive results for FVIII immunoglobulin G (IgG) antibodies in enzyme-linked immunosorbent assays (ELISA). This necessitates follow-up testing with more specific functional assays to accurately diagnose FVIII inhibition [8]. Additionally, Jacobs et al. highlighted the challenge of differentiating between LA and FVIII inhibitors, as both can prolong APTT and interfere with each other's detection [6]. Therefore, in patients with SLE and AIHA, it is crucial to use a combination of assays, including rotational thromboelastometry (ROTEM) and thrombin generation assay (TGA), to accurately differentiate between LA and FVIII inhibitors and avoid misinterpretation of test results [6].

The prognosis of AIHA is largely dependent on the promptness of diagnosis and treatment, as well as treating any existing comorbidities. AIHA management requires a dual approach: controlling acute bleeding and eradicating the FVIII inhibitors. Hemostatic control includes recombinant activated factor VII (rFVIIa) and aPCC as the preferred agents for controlling acute bleeding episodes in AIHA, especially in patients with high-titer inhibitors. These agents bypass the need for FVIII and are effective in achieving hemostasis [9,10]. Patients with low-titer inhibitors may benefit from recombinant human FVIII [11]. Immunosuppressive therapy is also crucial to eliminate the FVIII inhibitors. High-dose corticosteroids, such as intravenous methylprednisolone, are first-line therapy in most patients, with the addition of cyclophosphamide, demonstrating an increased likelihood of reaching stable and complete remission than with corticosteroids alone [11]. In refractory cases, rituximab has been successful in eradicating FVIII inhibitors [12]. Additional interventions such as plasmapheresis to rapidly reduce circulating inhibitor levels and intravenous immunoglobulin (IVIG) as adjunctive therapy may have a role, though less defined than the aforementioned treatments [11,13]. 

According to the Surveillance des Auto antiCorps au cours de l'Hémophilie Acquise (SACHA) registry, a French prospective study of 82 patients with AIHA, immunosuppressive therapy achieved complete remission in 61% of patients at three months and a 98% complete remission by one year [14]. Despite these interventions, the SACHA registry reports a significant all-cause mortality of 33% for patients with AIHA [14]. The leading cause of mortality in this study was sepsis secondary to infection, followed by bleeding. To understand this, one must examine the multiple pitfalls of lengthy immunosuppressive treatment and complications of unstable hemostasis. 

Immunosuppressive therapy, a necessity for FVIII inhibitor eradication, predisposes patients to infection. For example, a Dutch cohort study discovered the risk of infection is significantly increased in patients on steroid combination therapy (38.7%) versus steroids alone (10.6%) [15]. This risk is particularly pronounced in the elderly, who may have a compromised immune system [16]. Furthermore, the management of AIHA is complicated by patients with polypharmacy and underlying comorbidities that may mask or exacerbate symptoms [17]. The authors witnessed the reality of this scenario in this 73-year-old female patient with type 2 diabetes mellitus and cardiovascular disease who was readmitted shortly after discharge with neutropenic fever, likely secondary to cyclophosphamide-induced myelosuppression, requiring discontinuation of the offending drug and broad-spectrum antimicrobial therapy. Corticosteroids, the mainstay of treatment in AIHA, carry an increased likelihood of exacerbating comorbid conditions like T2DM, as well as an increased risk of osteoporosis, hypertension, and GI bleeding, among others [18]. 

AIHA, as the authors have described, involves the body’s immune system attacking and hemolyzing red blood cells, resulting in their destruction. As with other autoimmune disorders, this inflammation is not localized but rather systemic, affecting not only the blood vessels in which the blood cells are being sheared but the entire body. One place this can become apparent is the central nervous system, manifesting as anxiety, mood disorders, and even psychosis. Benros et al. demonstrated that autoimmune diseases and severe infections are significant risk factors for mood disorders. Specifically, a prior hospital contact for an autoimmune disease increases the risk of a subsequent mood disorder diagnosis by 45% [19]. In addition, the physical burden and chronic stress of managing a severe autoimmune condition like AIHA can contribute to psychiatric symptoms. The presence of systemic inflammation and the production of brain-reactive antibodies can further exacerbate psychiatric conditions. For example, Shimo et al. found that social stress can induce autoimmune responses against the brain, leading to increased levels of brain-reactive IgG antibodies, which are associated with depressive symptoms and social avoidance behavior [20]. These findings suggest that the persistent systemic inflammation from this patient’s autoimmune disorder may have contributed to the sudden onset of major depression and borderline catatonia despite no prior history of psychiatric illness.

## Conclusions

Despite significant advances in treatment, autoimmune hemolytic anemia (AIHA) remains a life-threatening condition associated with substantial morbidity and mortality. This case underscores the critical importance of prompt and accurate diagnosis, as delays can lead to severe complications, prolonged hospitalizations, and worsened prognoses. Managing AIHA presents a delicate balance between controlling hemolysis with immunosuppressive therapy while mitigating the risk of infection and excessive bleeding, particularly in patients with concurrent disorders or predisposing factors. Furthermore, this case highlights the profound impact that serious illnesses can have on mental health, emphasizing the need for psychological support alongside medical management. Given the complexity and variability of AIHA, a multidisciplinary approach involving hematologists, immunologists, and mental health professionals is essential to optimizing patient care. Early recognition, personalized treatment strategies, and ongoing research into novel therapeutic approaches are crucial to improving both survival rates and quality of life for individuals affected by this rare but serious disease.

## References

[1] Shetty S, Bhave M, Ghosh K (2011). Acquired hemophilia A: diagnosis, aetiology, clinical spectrum and treatment options. Autoimmun Rev.

[2] Knöbl P (2018). Prevention and management of bleeding episodes in patients with acquired hemophilia A. Drugs.

[3] Poston JN, Kruse-Jarres R (2022). Advances in acquired hemophilia A. Transfus Med Rev.

[4] Ellsworth P, Chen SL, Jones LA, Ma AD, Key NS (2025). Acquired hemophilia A: a narrative review and management approach in the emicizumab era. J Thromb Haemost.

[5] Gupta D, Chatterjee T, Sharma A, Ganguli P, Das S, Sharma S (2014). Rare case of acquired haemophilia and lupus anticoagulant. Indian J Hematol Blood Transfus.

[6] Chikasawa Y, Amano K, Shinozawa K (2023). Comprehensive comparison of global coagulation assays to differentiate lupus anticoagulant from acquired hemophilia A in patients with prolonged APTT. Int J Hematol.

[7] Jacobs JW, Gisriel SD, Iyer K, Rinder HM (2022). Concomitant factor VIII inhibitor and lupus anticoagulant in an asymptomatic patient. J Thromb Thrombolysis.

[8] Sahud M, Zhukov O, Mo K, Popov J, Dlott J (2012). False-positive results in ELISA-based anti FVIII antibody assay may occur with lupus anticoagulant and phospholipid antibodies. Haemophilia.

[9] Collins PW (2011). Management of acquired haemophilia A. J Thromb Haemost.

[10] Baudo F, Collins P, Huth-Kühne A (2012). Management of bleeding in acquired hemophilia A: results from the European Acquired Haemophilia (EACH2) Registry. Blood.

[11] Shen P, Li J, Tu S, Chen G, Chen C (2020). Acquired hemophilia A in a woman with systemic lupus erythematosus: a case report and review of literature. Medicine (Baltimore).

[12] Rungjirajittranon T, Suwanawiboon B, Nakkinkun Y (2024). First-line immunosuppressive therapies for acquired hemophilia A: a 25-year cohort experience and network meta-analysis. Thromb Res.

[13] Porru G, Mura V, Piga M (2008). Hemarthrosis as acute presentation of acquired hemophilia in a patient with systemic lupus erythematosus: successful treatment and long-lasting remission. Clin Rheumatol.

[14] Borg JY, Guillet B, Le Cam-Duchez V, Goudemand J, Lévesque H (2013). Outcome of acquired haemophilia in France: the prospective SACHA (Surveillance des Auto antiCorps au cours de l'Hémophilie Acquise) registry. Haemophilia.

[15] Schep SJ, van Dijk WE, Beckers EA (2021). Treatment of acquired hemophilia A, a balancing act: results from a 27-year Dutch cohort study. Am J Hematol.

[16] Barcellini W, Fattizzo B, Zaninoni A (2014). Clinical heterogeneity and predictors of outcome in primary autoimmune hemolytic anemia: a GIMEMA study of 308 patients. Blood.

[17] Barcellini W, Fattizzo B, Cortelezzi A (2018). Autoimmune hemolytic anemia, autoimmune neutropenia and aplastic anemia in the elderly. Eur J Intern Med.

[18] Barcellini W, Fattizzo B (2021). How I treat warm autoimmune hemolytic anemia. Blood.

[19] Benros ME, Waltoft BL, Nordentoft M, Ostergaard SD, Eaton WW, Krogh J, Mortensen PB (2013). Autoimmune diseases and severe infections as risk factors for mood disorders: a nationwide study. JAMA Psychiatry.

[20] Shimo Y, Cathomas F, Lin HY (2023). Social stress induces autoimmune responses against the brain. Proc Natl Acad Sci U S A.

